# Design and experiment of Panax notoginseng root orientation transplanting device based on YOLOv5s

**DOI:** 10.3389/fpls.2024.1325420

**Published:** 2024-03-08

**Authors:** Qinghui Lai, Yongjie Wang, Yu Tan, Wenqiang Sun

**Affiliations:** ^1^ School of Energy and Environment Science, Yunnan Provincial Rural Energy Engineering Key Laboratory, Yunnan Normal University, Kunming, China; ^2^ Faculty of Modern Agriculture Engineering, Kunming University of Science and Technology, Kunming, China

**Keywords:** *Panax notoginseng*, target detection, machine vision, orientation transplanting, YOLOv5

## Abstract

Consistent root orientation is one of the important requirements of *Panax notoginseng* transplanting agronomy. In this paper, a *Panax notoginseng* orientation transplanting method based on machine vision technology and negative pressure adsorption principle was proposed. With the cut-main root of *Panax notoginseng* roots as the detection object, the YOLOv5s was used to establish a root feature detection model. A *Panax notoginseng* root orientation transplanting device was designed. The orientation control system identifies the root posture according to the detection results and controls the orientation actuator to adjust the root posture. The detection results show that the precision rate of the model was 94.2%, the recall rate was 92.0%, and the average detection precision was 94.9%. The Box-Behnken experiments were performed to investigate the effects of suction plate rotation speed, servo rotation speed and the angle between the camera and the orientation actuator(ACOA) on the orientation qualification rate and root drop rate. Response surface method and objective optimisation algorithm were used to analyse the experimental results. The optimal working parameters were suction plate rotation speed of 5.73 r/min, servo rotation speed of 0.86 r/s and ACOA of 35°. Under this condition, the orientation qualification rate and root drop rate of the actual experiment were 89.87% and 6.57%, respectively, which met the requirements of orientation transplanting for *Panax notoginseng* roots. The research method of this paper is helpful to solve the problem of orientation transplanting of other root crops.

## 1 Introduction

*Panax notoginseng*, a highly regarded Chinese medicinal herb, holds significant medicinal and economic value within China (Gao et al., 2016; Gao et al., 2022; Thorpe et al., 2023). In the cultivation process of *Panax notoginseng*, the transplanting of roots plays a pivotal role. To ensure optimal growth conditions, it is crucial to plant *Panax notoginseng* roots in a uniform orientation (Lai et al., 2021). This uniformity promotes efficient absorption of light and nutrients, leading to improved root survival rates. Despite the importance of root orientation, most current automatic transplanting machines lacking this capability. The orientation of *Panax notoginseng* roots mainly relies on manual labour to complete (Qin et al., 2022). The low efficiency and high cost of manual work constrain the long-term development of *Panax notoginseng* industry. Therefore, solving the problem of automatic orientation transplanting of *Panax notoginseng* roots is of great significance for improving the level of mechanisation of *Panax notoginseng* and the economic benefits of the industry.

Many scholars have conducted some researches on the orientation of crops (Li et al., 2020; Cheng et al., 2023; Zhu et al., 2023). In the orientation of regularly shaped crops, Hou et al. (2020) and Geng et al. (2018) designed a double duckbill orientation device and a three-stage conical hopper orientation device to achieve consistent tail orientation of garlic. These devices were oriented based on the principle that the centre of gravity of cloves is close to the tail and were suitable for regular shaped garlic. Cui et al. (2021) proposed a conical roller kale orientation device based on the principle of moment of inertia. Under the support force and friction force of the rollers, the kale rotates along the central axis with the smallest moment of inertia to achieve automatic orientation of the kale. However, *Panax notoginseng* root is an irregularly shaped crop, although it has obvious centre of gravity characteristics. Most of the roots have curved roots and are easily damaged. Therefore, these devices may not be suitable for transplanting *Panax notoginseng* roots. In the previous research of *Panax notoginseng* root transplanting, our team successively developed a double suction hole orientation transplanting device and a root guiding tube transplanting device by using air suction force and centrifugal force, and achieved good performance in orientation transplanting (Lai et al., 2021; Qin et al., 2022). Unfortunately, both devices have the problem of low efficiency. Furthermore, the mechanical orientation process itself entails unavoidable collisions and friction, which in turn results in surface damage to the roots.

With the emergence of machine vision technology in the agricultural field (Chen et al., 2002; Makky and Soni, 2013; Sofu et al., 2016), it provides a new idea for efficient orientation. San et al. (2021) used image processing Harris corner point detection algorithm to identify the head and tail of the fish, combined with the adjustment device to achieve the head and tail orientation of the fish body. Ai et al. (2022) identified the head and tail orientation of the fish body based on Resnet-18 classification network, identified the ventral and dorsal orientation of the fish body through image processing, and design an orientation and finishing device for the fish body. Li et al. (2020) proposed a multi-feature algorithm for effectively identifying the orientation of garlic in the image. The algorithm has higher accuracy than single-feature recognition methods for garlic varieties with large differences in features. The above recognition method is mainly based on traditional image processing, which recognizes the posture based on colour or shape features. Such methods are greatly disturbed by light and environmental background and have low robustness. The YOLO family of algorithms, as excellent deep learning models with high detection capability and low cost, have been widely used in crop recognition such as pineapple and cherry (Meng et al., 2023). Wang et al. (2023) developed an image algorithm for recognising lychee picking points using YOLOv8-Seg model. Hou et al. (2023) proposed an improved YOLOv7 model for recognising occluded cherry tomato. Therefore, the YOLO model will be used in this paper for identifying *Panax notoginseng* root features for fast and accurate orientation operations.

To solve the problem of automatic root orientation during *Panax notoginseng* root transplanting, this paper incorporated machine vision technology and orientation control system based on existing research. The transplanting quality and performance of the device proposed in this paper was significantly improved as compared to the existing transplanting devices. By adjusting the working parameters and picture databases, it enables efficient orientation transplanting of other root crops. The main contributions are summarized as follows:

(1) Combining machine vision technology and negative pressure adsorption principle, an efficient and low-loss method for orientation transplanting of *Panax notoginseng* roots was proposed.
(2) A *Panax notoginseng* root feature detection model was proposed based on the YOLOV5s model.
(3) A control method for vision-based automatic orientation of *Panax notoginseng* roots was developed.
(4) The optimal operating parameters of the orientation transplanting device for *Panax notogiseng* roots were obtained by Box-Behnken experiment.

## 2 Materials and methods

### 2.1 Shape parameters of Panax notoginseng root

**Figure 1:**
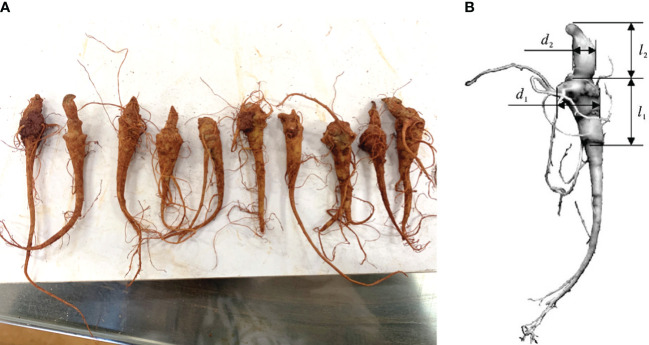
The parameter diagram of *Panax notoginseng* root. **(A)** Schematic diagram of the appearance of the roots; **(B)** Schematic diagram of key parameters.

In this paper, annual *Panax notoginseng* roots produced in Wenshan, Yunnan Province were chosen as the research object. A total of 300 *Panax notoginseng* roots were randomly selected for a measurement experiment. The main root length (*l*_1_), main root diameter (*d*_1_), cut length (*l*_2_), and cut diameter (*d*_2_) of each root were measured using a vernier calliper (Lai et al., 2018). The parameter diagram of the *Panax notoginseng* root is presented in **Figure 1**, and the measurement results are summarized in **Table 1**.

**Table 1:** Size parameters of Panax notoginseng root.

Index	*l*_1_/mm	*d*_1_/mm	*l*_2_/mm	*d*_2_/mm
Average value	22.42	12.64	13.28	6.15
Maximum value	26.12	15.68	17.74	8.89
Minimum value	17.50	9.08	8.38	4.35

*l*_1_ is the main root length; *d*_1_ is the main root diameter; *l*_2_ is the cut length; *d*_2_ is the cut diameter.

### 2.2 Overall structure and working principle

**Figure 2:**
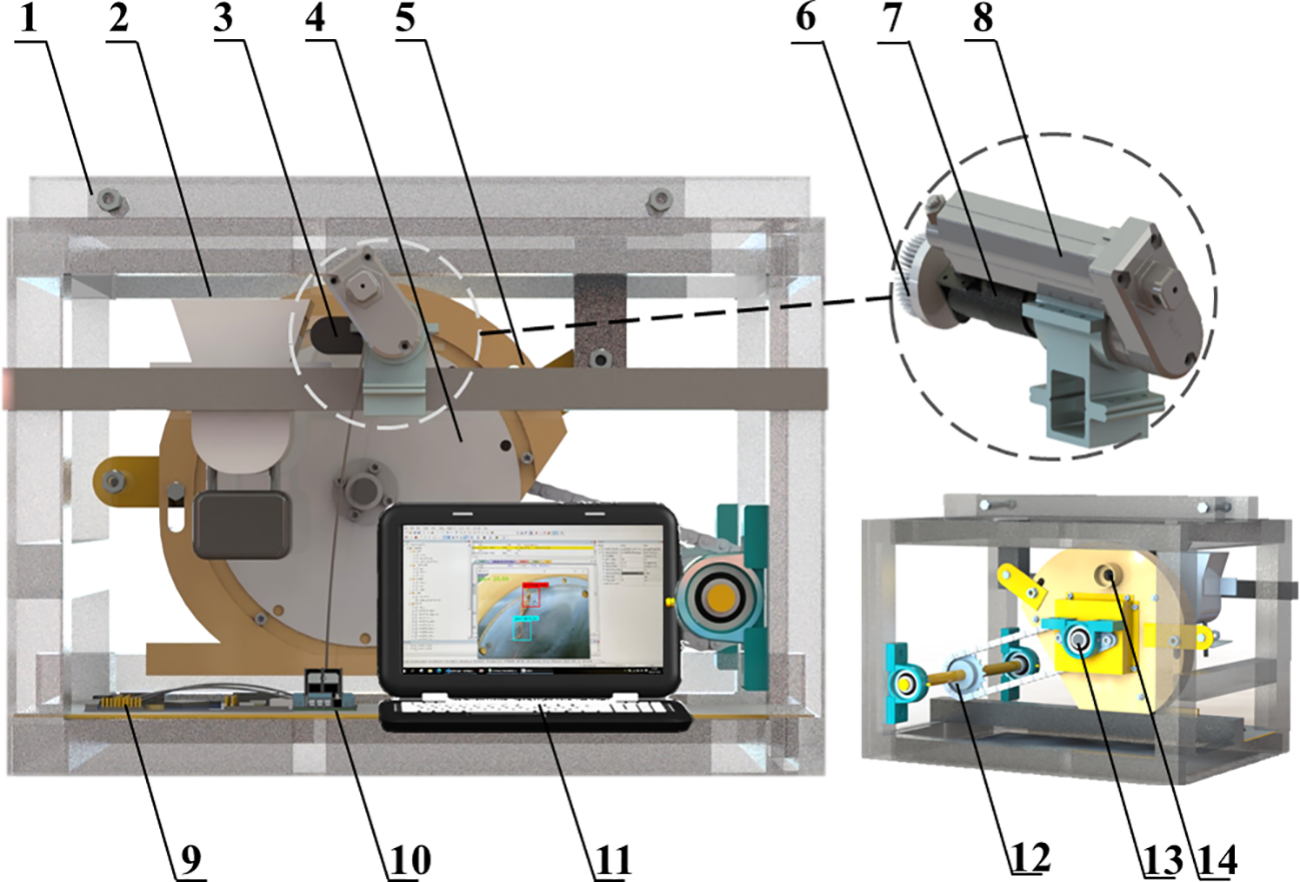
Schematic diagram of the structure of the transplanting device. 1. machine frame; 2. root box; 3. depth camera; 4. suction plate; 5. main shell; 6. brush; 7. SG90 servo; 8. linear actuator; 9. STM32; 10. L298N driver module; 11. PC; 12 transmission mechanism; 13. transmission shaft; 14. airflow inlet.

The *Panax notoginseng* root orientation transplanting device consists of machine frame, root suction device, orientation actuator and orientation control system, as shown in **Figure 2**. The root suction device mainly includes root box, suction plate, main shell, airflow inlet and transmission shaft; the orientation actuator mainly includes linear actuator, brush and SG90 servo; the orientation control system mainly includes PC, STM32, L298N driver module and depth camera (Intel, D435). The orientation actuator is mounted on the upper part of the suction plate by a connection frame. The orientation actuator is adjusted to a position with a reasonable distance between the suction hole and the orientation actuator. The backside of the suction plate fits tightly with a sealing air cushion. A vacuum fan is connected to the airflow inlet to generate negative pressure in the air chamber. The motor rotates the suction plate by the transmission shaft.

**Figure 3:**
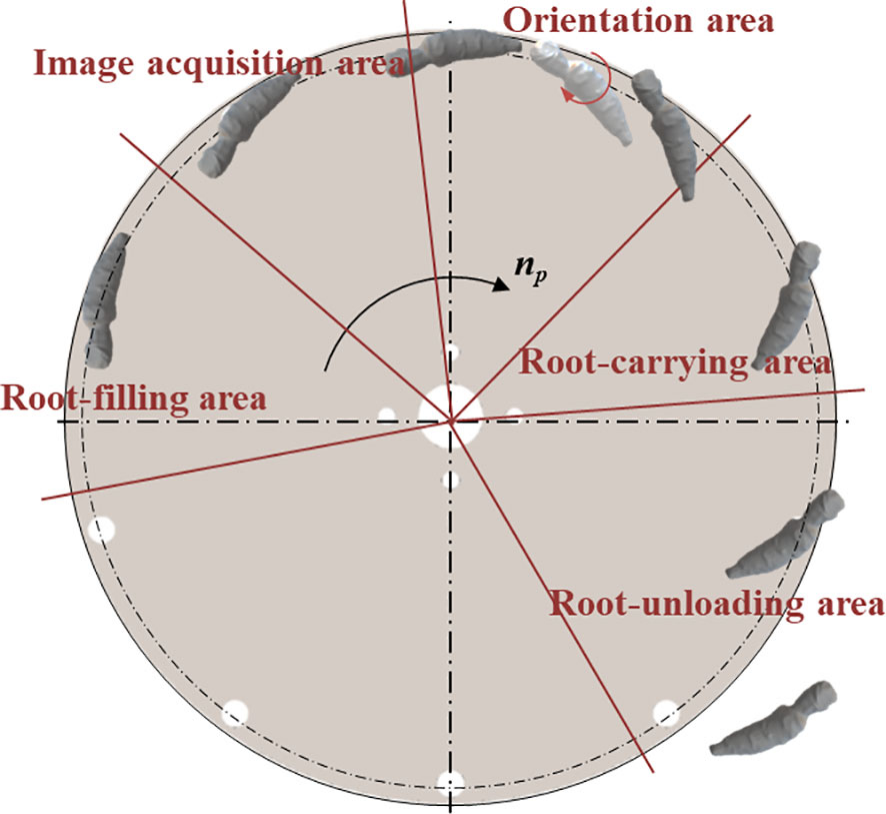
Schematic representation of working area division of the transplanting device.

**Figure 4:**
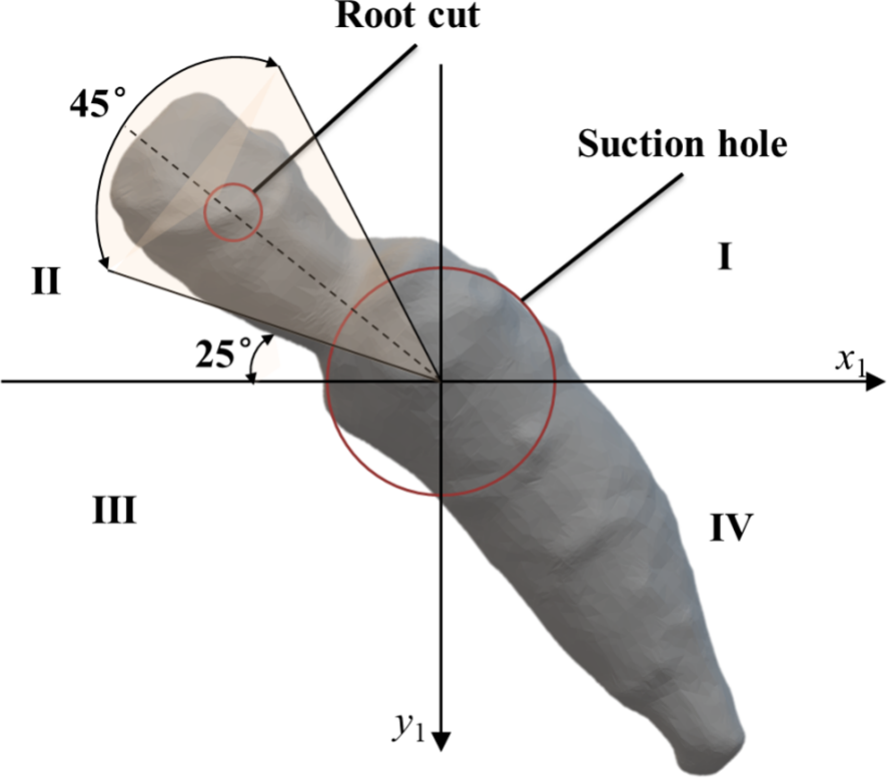
Schematic diagram of the ideal posture of the root.

The whole working process can be divided into five areas, as shown in **Figure 3**, including root-filling area, image acquisition area, orientation area, root-carrying area and root-unloading area. When the transplanting device works, the roots in the root box are moved up and down by vibration. The suction holes on the suction plate adsorb the roots through negative airflow. The motor drives the transmission shaft to control the rotating of the suction plate. When the root reaches the image acquisition area, the camera collects the image information of the root and transmits the image information to the PC The PC processes the image and sends the position information of the cut and main root to the STM32; the STM32 determines the current root posture based on the position of the cut; subsequently, the root’s orientation adjustment instruction is sent to the orientation actuator through the control circuit. The orientation actuator performs corresponding orientation actions for roots with different postures. Specifically, the brush is driven by linear motor to press the root on the suction plate, and then the brush is rotated by servo control at the corresponding angle as programmed, and finally the actuator is reset; after the completion of the orientation adjustment, the root maintains the ideal posture(as shown in **Figure 4**) to pass through the root-carrying area and the root unloading area; the negative pressure airflow disappears in the unloading area, and the root is discharged under the action of gravity and centrifugal force.

### 2.3 Design of key components

#### 2.3.1 root suction device

The root suction device is the key component for realising the low damage transport of roots. Roots in the root box are adsorbed by means of a negative air flow. This method is less damaging to the roots and can be adapted to different shapes.

The structural parameters of the suction plate directly affect the root filling performance (Xu et al., 2023). In general, the longer the residence time of the suction holes in the root-filling area, the more favourable the root filling. The passage time *t*_0_ of any suction hole in the root filling area can be expressed as:


(1) $$t_{0}=\frac{L_{0}}{v_{0}}=\frac{\frac{10^{3}Dr}{2}}{\frac{10^{3}D\pi n_{p}}{60}}=\frac{30r}{\pi n_{p}}$$

where *L*_0_ is the arc length of the root-filling area, m; *v*_0_ is the linear velocity at the suction hole, m/s; *D* is the suction plate diameter, mm; *n_p_* is the rotation speed of the suction plate, r/min; *r* is the arc degree of the root-filling area, rad.

According to (Equation 1), the passage time *t*_0_ of the suction hole in the root area is related to the arc length of the root-filling area and the suction plate rotation speed, but not related to the suction hole diameter. Therefore, the main factors affecting the suction plate diameter are the size of the root, the volume of the device and the operating speed. In the existing studies, the suction plate diameter of the suction device is generally 140-260 mm (Gao et al., 2023). Due to the large size of *Panax notoginseng* roots, an appropriate increase in the suction plate diameter is favourable for the filling of the roots. Considering the above factors, the suction plate diameter *D* was selected as 250 mm.

The suction hole diameter has a significant effect on the filling quality. When the negative pressure is constant, the suction hole diameter increases to make the filling capacity decrease; the suction hole diameter becomes smaller, then the suction force per unit area increases. However, the root was not adsorbed stably due to the small negative pressure area. In order to facilitate processing, the type of suction hole is selected as a cylindrical hole. Referring to the design formula of the suction hole of the air-suction seed-metering device (Equation 2), the suction hole diameter is determined as *d* = 8.6 mm.


(2) $$d=(0.6\sim 0.7)d_{1}$$

**Figure 5:**
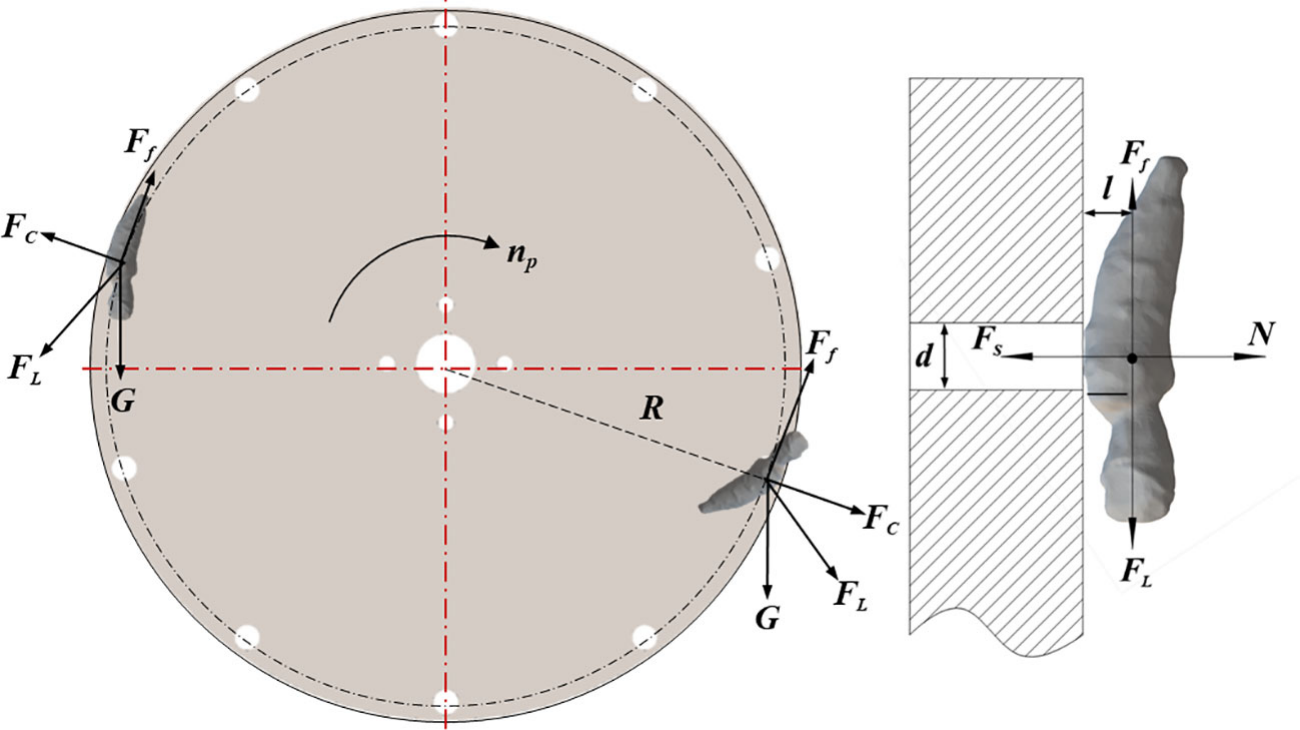
Force analysis of root during adsorption process.

When the suction hole passes through the root filling zone, the root is captured by the negative pressure airflow generated by the fan. The roots adsorbed on the suction hole pass through the orientation area, root carrying area and root unloading area in turn to complete the transplanting operation. The force analysis of the root in the adsorption state is carried out by taking a single root adsorbed by the suction hole as the research object; and assuming that the root is a rigid body of homogeneous material, the friction and collision between the root and the suction plate are not considered. The force analysis is shown in **Figure 5**.

Based on the theory of fluid mechanics and rigid body dynamics, the force equation of the root was established as in Equation 3:


(3) $$\{\begin{array}{l} \begin{array}{l} \left(F_{s}-N\right)\frac{d}{2}=F_{L}\cdot l\\ F_{L}=G+F_{c} \end{array}\\ H=\sqrt{G^{2}+F_{c}^{2}+2GF_{c}\cos\alpha }\\ F_{c}=mR\omega^{2}\\ \begin{array}{l} P_{0}=\frac{F_{s}}{S}\\ S=\frac{\pi d^{2}}{4} \end{array} \end{array}$$

where *F*_s_ is the suction force on the root, N; *N* is the support force of the suction plate on the root, N; *l* is the distance between the centre of gravity of the root and the suction plate, mm; *d* is the suction hole diameter, mm; *F*_L_ is the combined force of the root’s gravity and the centrifugal force, N; *α* is the angle between gravity and centrifugal force; *ω* is the angular velocity of the suction plate, rad/s; *r* is the distance between the centre of the suction hole and the centre of the plate, mm; *P*_0_ is the negative pressure provided by the fan, kPa; *S* is the cross-sectional area of the suction hole, mm^2^.

If *N*=0, the negative pressure *P*_0_ provided by the fan can be expressed as:


(4) $$P_{0}=\frac{8l\left(\sqrt{G^{2}+F_{c}{\;}^{2}+2GF_{c}\cos\alpha }\right)}{\pi d^{3}}$$

During the operation of the device, the adsorption status of the root is influenced by its own conditions (sealing between the gas chamber and the suction plate) and objective conditions (vibrations generated by the drive shaft). Therefore, the actual required negative pressure should be multiplied by the suction reliability coefficient and the operational reliability coefficient. According to Equation 4, the required negative pressure is maximum when cos*α* = 1, i.e., when gravity is in the same direction as the centrifugal force. The minimum negative pressure *P*_t_ which satisfies the adsorption of a single root is expressed as in Equation 5:


(5) $$P_{t}=\frac{8K_{1}K_{2}l}{\pi d^{3}}\left(G+F_{c}\right)$$

where *K*_1_ is the reliability coefficient of root suction, taking 1.8~2.0; *K*_2_ is the reliability coefficient of orientation transplanting device operation, taking 1.6~2.0.

#### 2.3.2 Design of the orientation control system

**Figure 6:**
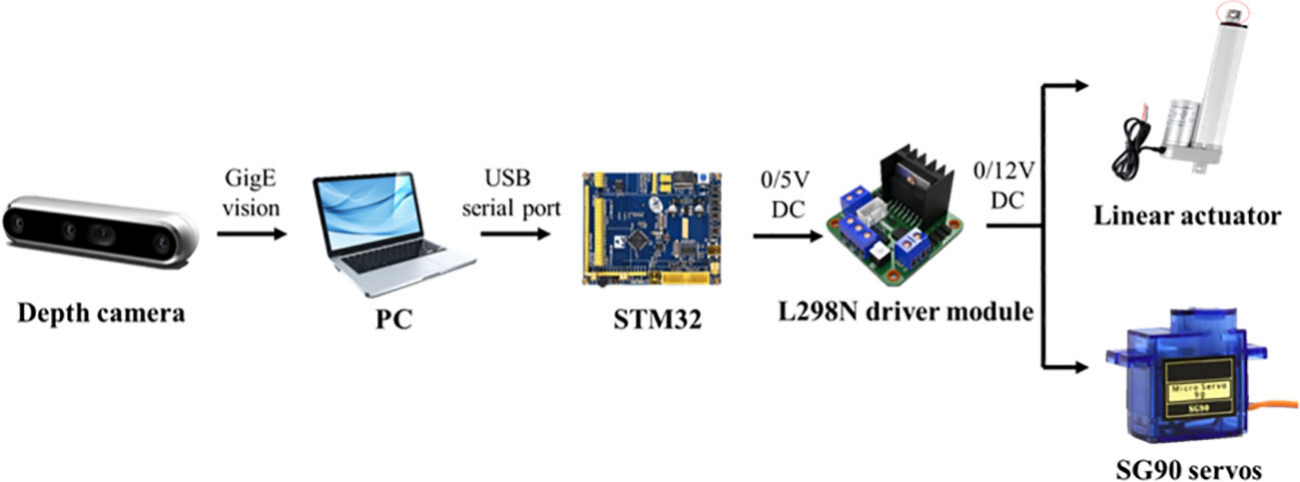
Orientation control system composition.

The orientation control system is used to complete the acquisition of root images, the identification of root posture and orientation of root during transplanting of *Panax notoginseng* roots. Orientation control system components are shown in **Figure 6**.

**Figure 7:**
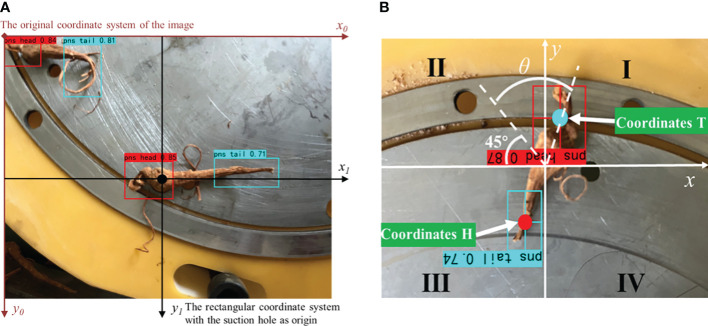
Flow of root posture recognition. **(A)** coordinate conversion; **(B)** servo rotation angle.

**Figure 8:**
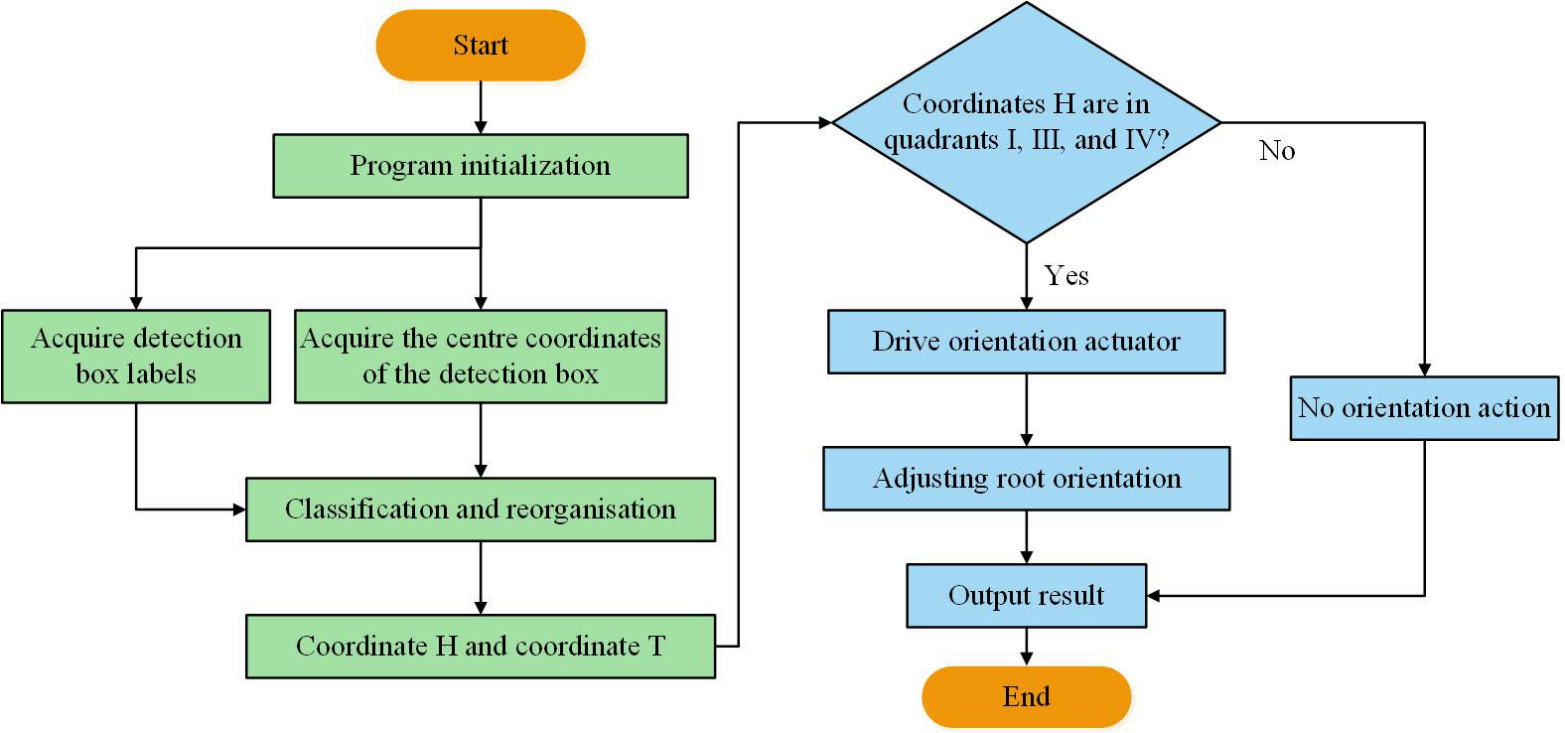
Flow chart of the control program of the orientation control system.


**Figure 9:**
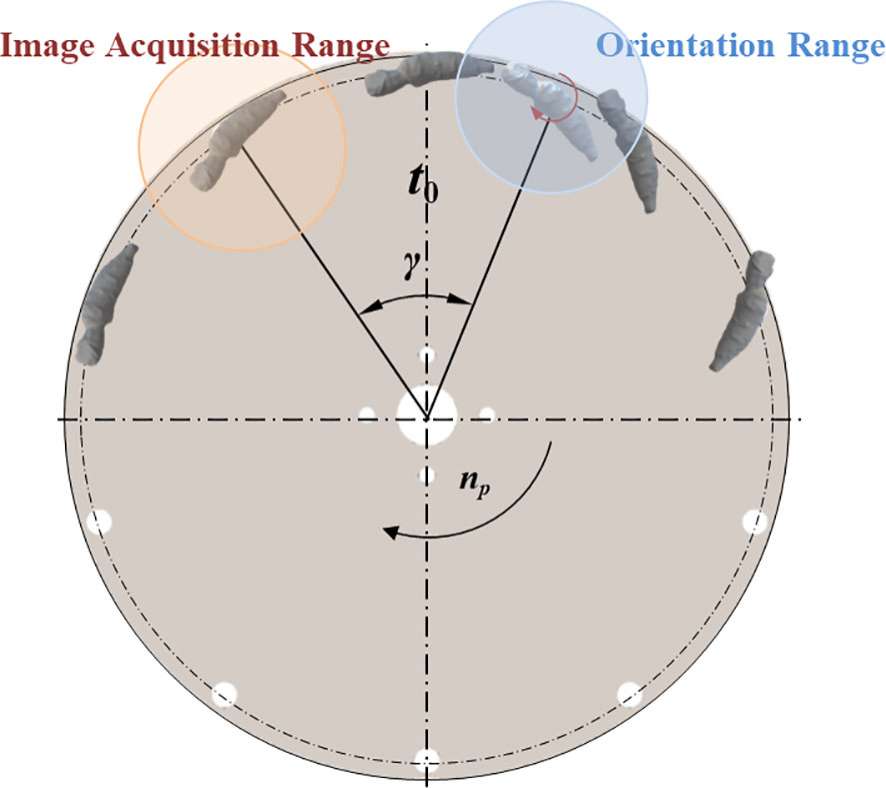
Schematic diagram of angle between the camera and orientation actuator.

The orientation control system is mainly composed of camera, PC, target detection programme, STM32, L298N driver module, linear actuator, SG90 servo, power supply,etc. When the root enters the image acquisition area, the camera collects the image of the root and transmits it to the PC; the detection program in the PC identifies the position information of the cut and main root, and transmits the identification result to the STM32. STM32 determines the current root posture based on the cut position and controls the orientation actuator to adjust the cutting direction. The whole process is divided into the following 5 steps:

(1) The target detection algorithm in the PC identifies the cuttings feature and the main root feature of *Panax notoginseng* roots. Extract the centre coordinates of the prediction frame, i.e. extract the cut coordinates H and the main root coordinates T.
(2) Root coordinates are input to the STM32 through the serial port, The STM32 uses the DMA method to filter and eliminate the duplicate coordinates received. In the filtered coordinate group, the plural of the coordinate value is selected as the actual coordinate value of the root.
(3) Convert the coordinate system where the root is located from the original coordinate system of the image to the rectangular coordinate system with the suction hole as origin, as shown in **Figure 7A**.
(4) Mark the root posture according to the quadrant where the cut is located. Then, calculate the angle between the current posture and the second quadrant angular bisector, as shown in **Figure 7B**.
(5) After receiving the orientation command, the linear motor drives the brushes to approach the root. The servo rotates the angle calculated by the STM32 to adjust the root posture.

when the root enters the orientation area, the STM32 controls the orientation actuator to complete the corresponding orientation action based on the posture of the root. The signal delay timing of the orientation action is determined by the angle between the camera and the orientation actuator(ACOA) and the suction plate rotation speed, which can be calculated by Equation 6. The control program flow of the orientation control system is shown in **Figure 8**.

(6) $$t_{0}=\frac{\alpha_{0}\pi D}{360v_{0}}-\frac{l_{0}}{v_{t}}$$

where *t*_0_ is the actuation delay time, s; *α*_0_ is the ACOA, °, as shown in **Figure 9**; *D* is the suction plate diameter, mm; *v*_0_ is the suction hole linear velocity, m/s; *l*_0_ is the distance between the suction plate and the orientation actuator, mm; *v_t_* is the movement speed of the linear actuator.

### 2.4 *Panax notoginseng* root feature detection model based on YOLOv5s

#### 2.4.1 *Panax notoginseng* root image preprocessing

**Figure 10:**
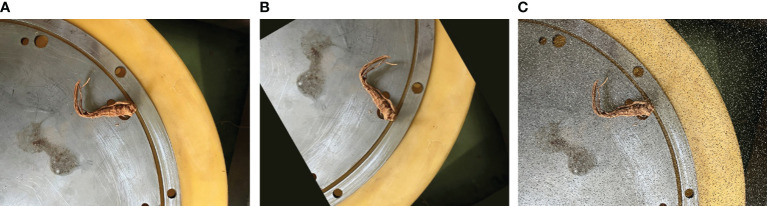
Processed image dataset. **(A)** original images; **(B)** rotation and cutting; **(C)** adding Gaussian noise.

300 roots were selected for root filling test, and 1200 images of *Panax notoginseng* roots in random posture were collected. The images were obtained in pixel sizes of 4032 × 3024. To improve the generalisation ability of the root posture recognition model, the original images were preprocessed. The total number of images in the dataset was expanded to 2500 by brightness enhancement, random flipping and Gaussian noise processing, as shown in **Figure 10**. The constructed root image dataset was labelled using LabelImg, as shown in **Supplementary Figure 1**. By randomly dividing the labelled root image dataset into two sets with an 8:2 ratio, 2000 images in the training set, 500 in the test set were obtained.

#### 2.4.2 YOLOv5s detection model

**Figure 11:**
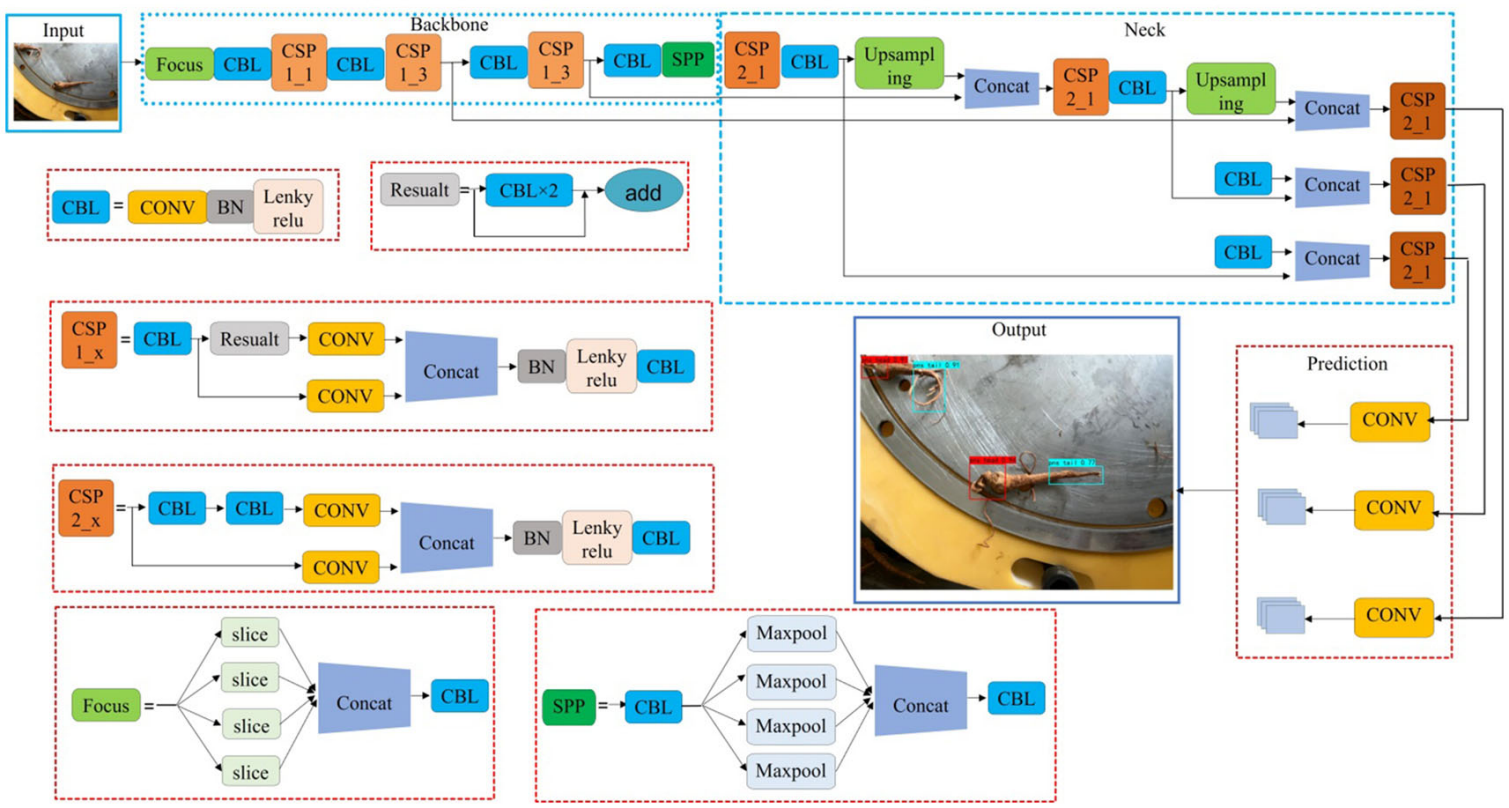
Labeling interface.

Accuracy, real-time and lightweight of root feature detection model are the key to identify the *Panax notoginseng* root posture.YOLOv5, as a single-stage target detection algorithm, has faster speed, better accuracy and smaller model size compared with its previous versions (Zhang P. et al., 2022; Tan et al., 2023; Zhao et al., 2023). Therefore, in this study, YOLOv5s model was selected for root feature recognition to achieve efficient detection of root cut and main root. The structure of the model includes four parts: input, backbone, neck and head (Zhang B. et al., 2022; Li et al., 2023; Xiao et al., 2023), as shown in **Figure 11**. The Backbone network is used to achieve the extraction of root features; the Neck network integrates the feature graphs of different scales to improve the detection performance; and the detection network obtains the root feature types, confidence level, and detection box position.

#### 2.4.3 Model training

The root characterisation recognition model was trained under Windows 10 operating system. The system CPU is Intel XeonW-214, GPU is NVIDIA RTX2080Ti(11GB), and the RAM is 64G.The deep learning framework is Pytorch 11.6, and the software environment is Python 3.9, cuda11.6. **Supplementary Figure 2** shows the training loss curve for the YOLOv5s model. As the number of iterations increases, the total loss of the model essentially converges to a stable value.

#### 2.4.4 Model evaluation indexes

The model evaluation indexes include precision, recall, F1, and mAP, as shown in Equations 7–10.


(7) $$\text{Precision}=\frac{TP}{TP\;+\;FP}\times 100\%$$

(8) $$Recall=\frac{TP}{TP\;+\;FN}\times 100\%$$

(9) $$F_{1}=\frac{2\times \text{Precision}\times Recall}{\text{Precision }+\;Recall}\times 100\%$$

(10) $$mAP=\frac{1}{N}\sum_{i=1}^{N}AP_{i}\times 100\%$$

where *TP* is the total number of correct predictions for positive samples, *FP* is the total number of incorrect predictions for positive samples, *FN* is the total number of incorrect predictions for negative samples, *AP_i_* is the detection accuracy of category *i*, and *N* is the number of categories.

#### 2.4.5 Comparison of recognition results of different detection models

In order to objectively evaluate the performance of the selected models, YOLOv5s, YOLOv5x, Faster R-CNN, and SSD network models were selected to conduct comparative experiments on the test set; using precision, recall, and average precision value as the evaluation indexes, the performance comparison of each network model is shown in **Table 2**.

**Table 2:** Comparison of detection results of different target detection algorithms.

Model	Precision/%	Recall/%	F_1_/%	mAP/%
YOLOv5s	94.2	92.0	93.1	94.9
YOLOv5x	91.0	91.4	91.2	91.4
Faster R-CNN	90.2	91.7	90.9	92.9
SSD	73.4	72.6	73.0	73.7

As shown in **Table 2**, the mAP of each detection model reaches over 91% except for the SSD model.YOLOv5s has the highest precision, recall, F_1_, and mAP, while the precision, recall, F_1_, and mAP values of YOLOv5x and Faster R-CNN are not much different.

**Figure 12:**
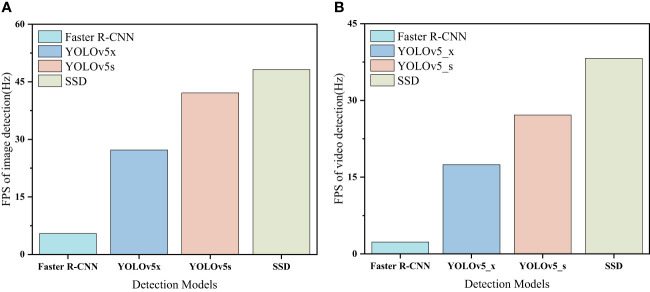
Network structure of root feature detection model.

The FPS is extremely important in the target detection process. Higher FPS means faster detection speed. Furthermore, the comparison of image FPS and video FPS for different detection models is shown in **Figure 12**. As shown in **Figure 12**, with the same hardware configuration, SSD can provide the fastest frame rate in image detection and video detection; YOLOv5 series provide about 35Hz in video detection; while Faster R-CNN detection is the slowest, and the average frame rate of video detection is only 3~5 Hz.

The operation of the orientation transplanting device is a continuous process. As the speed of operation increases, the number of roots passing the image acquisition area per unit of time gradually increases. This requires a higher detection speed for the detection model. The YOLOv5s has an obvious advantage over other algorithm models in detection speed with a high detection accuracy. Therefore, the YOLOv5s selected in this paper can achieve the fast real-time detection of the cuttings and main roots of *Panax notoginseng* roots.

### 2.5 Experimental scheme

The detection accuracy of the YOLOv5s model is the key to accurately identify the root posture. Whereas, the overall performance of the orientation transplanting device is determined by the working parameters. From the previous analysis, the suction plate rotation speed, the ACOA, and the servo rotation speed may affect the root orientation performance. Therefore, in this section, a single-factor experiment and Box-Behnken experiment were designed for analysing the effects of these factors on the orientation performance.

#### 2.5.1 Evaluation indexes and experimental equipment

When the orientation transplanting device is in operation, the root rotates with the suction plate to complete the orientation and root discharge work. During this process, due to the rapid rotation speed of the suction plate or the movement of the orientation actuator, the root may be detached from the suction hole and drop, resulting in failed orientation transplanting. Therefore, the orientation qualification rate and root drop rate were selected as evaluation indexes for the experiment, as shown in Equations 11, 12.


(11) $$W_{1}=\frac{W_{o}}{W_{all}}\times 100\%$$

(12) $$W_{2}=\frac{W_{d}}{W_{all}}\times 100\%$$

where *W*_1_ is the root orientation qualification rate, %; *W*_o_ is the number of roots qualified for orientation in each experiment, %; *W*_all_ is the number of roots in each experiment, %; *W*_2_ is the root drop rate, %; and *W*_d_ is the number of roots dropped from the suction plate in each experiment, %.

**Figure 13:**
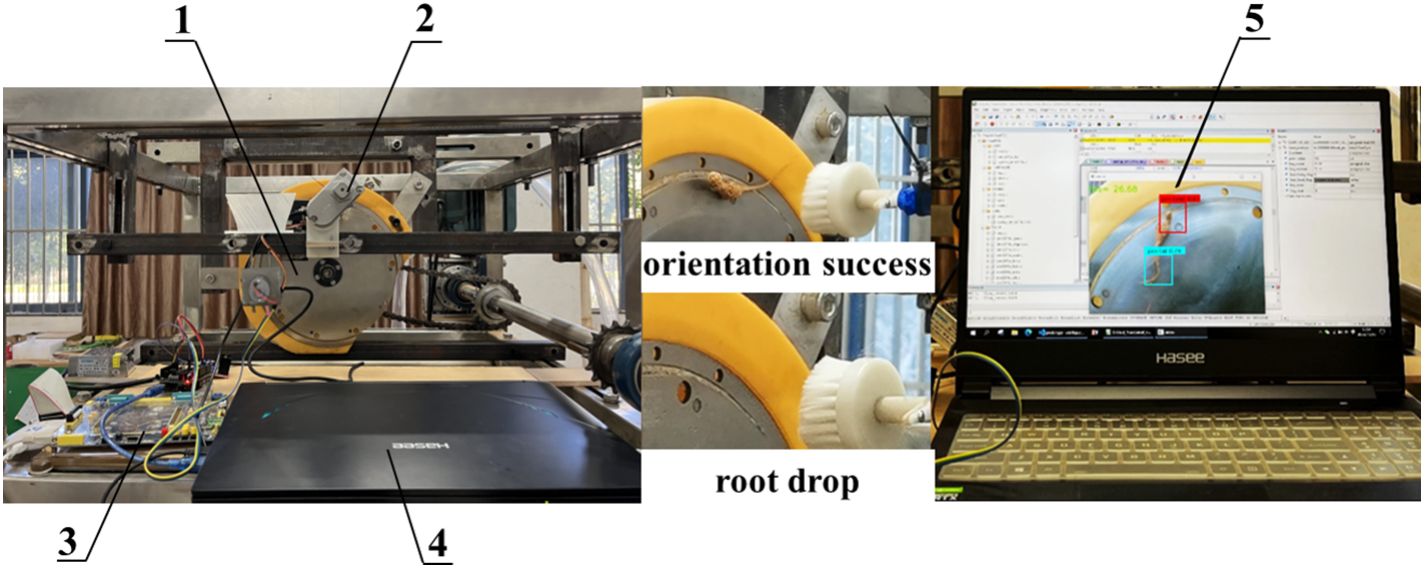
Training loss curve.

**Figure 13** shows custom-made orientation transplanting device for *Panax notoginseng* roots. The orientation transplanting device was installed in the JPS-12 seed arrangement test stand (Harbin Autobona Technology Co., Ltd., Harbin, China). The JPS-12 seed arrangement test stand provided the power source and negative airflow for the device.

#### 2.5.2 Single-factor experimental design

The suction plate rotation speed, the ACOA, and the servo rotation speed were selected as the experimental factors to carry out the single-factor experiment. The factor levels of the single-factor experiment are listed in **Table 3**. The orientation transplanting effect of 100 *Panax notoginseng* roots was continuously measured in each group of experiments. Each group of experiments was repeated three times, and the experimental results were averaged. Considering that the negative pressure was significant to the adsorption stability of roots, the negative pressure was set to 3 kPa to ensure that the roots were stably adsorbed on the suction plate.

**Table 3:** Levels of factors in the single-factor experiment.

Level	Factor		
	Suction plate rotation speed (r/min)	Servo rotation speed(r/s)	ACOA(°)
1	5.0	0.8	10
2	5.5	0.9	20
3	6.0	1.0	30
4	6.5	1.1	40
5	7.0	1.2	50

#### 2.5.3 Box-Behnken experimental design

In order to determine the optimum combination of operating parameters, A Box Behnken experiment was conducted to analyse the effect of suction plate rotation speed, ACOA and servo rotation speed on the indexes, based on the single-factor experiment. The level code table of experimental factors is shown in **Table 4**. The experimental programme and results are shown in **Supplementary Table 1**. Where *X*_1_, *X*_2_ and *X*_3_ are the coded values of the suction plate rotation speed, servo rotation speed and ACOA, respectively. The experimental indexes are orientation qualification rate *Y*_1_ and root drop rate *Y*_2_ respectively.

**Table 4:** Box-Behnken experimental factors of codes.

Factor	Code		
	-1	0	1
Suction plate rotation speed (r/min)	5	6	7
Servo Rotation speed(r/s)	0.8	0.9	1
ACOA(°)	20	30	40

## 3 Results

### 3.1 Results and analyses of the single-factor experiment

**Figure 14:**
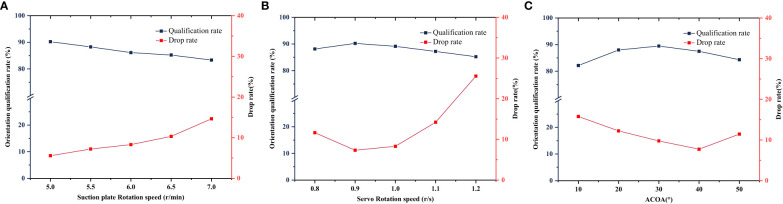
Results of single-factor experiments on **(A)** suction plate rotation speed, **(B)** servo rotation speed, **(C)** ACOA.

The single-factor experiment of suction plate rotation speed was carried out under the conditions that the suction plate rotation speed was 5, 5.5, 6, 6.5 and 7 r/min, the servo rotation speed was 1 r/s, and the ACOA was 30°. The experimental results are shown in **Figure 14A**. **Figure 14A** shows that with the increase of suction plate rotation speed, the orientation qualification rate decreases and the root drop rate increases. When the suction plate rotation speed was 7 r/min, the root drop rate reached 15.21% and the orientation qualification rate reached 90.23%. At this time, the centrifugal force on the root increased, and the root was easily detached from the suction hole. Meanwhile, the working time used for the orientation actuator decreased, and the root was not easy to be adjusted to the best posture.

The single-factor experiment of the servo rotation speed was carried out under the conditions that the servo rotation speed was 0.8, 0.9, 1, 1.1, 1. 2 r/s, the suction plate rotation speed was 6 r/min, and the ACOA was 30°. The experimental results are shown in **Figure 14B**. **Figure 14B** shows that the orientation qualification is firstly increases and then decreases with the increase of the servo rotation speed. When the servo rotation speed is 0.9 r/s, the orientation qualification index is the highest. This indicates that the increase of servo rotation speed in a certain range accelerates the orientation efficiency of the servo. And when the servo rotation speed is 1.2 r/s, due to the speeding up of the orienting action, the root is more disturbed by the brush and easily falls off. The root drop rate is highest.

The single-factor experiment of the ACOA was carried out under the conditions that ACOA was 10, 20, 30, 40, 50°, the suction plate rotation speed was 6 r/min, and the servo rotation speed was 1 r/s. The test results are shown in **Figure 14C**. **Figure 14C** shows that with the increase of the ACOA, the orientation qualification rate first increases and then decreases. When the ACOA is 30°, the orientation qualification rate reaches the highest, is 88.47%. At this time, when the root enters the orientation execution area, the orientation data of the root reaches the orientation actuator at the same time, and the orientation effect is optimal.

### 3.2 Results and analyses of Box-Behnken experiment

#### 3.2.1 Regression equation and significance analysis

To investigate the effects of the factors and their interactions on the experimental indices further, the experimental results were analysed by analysis of variance (ANOVA) using Design-expert (Stat-Ease, Minneapolis, Minnesota, USA). The ANOVA results are shown in **Supplementary Table 2**.

As shown in **Supplementary Table 2**, the suction plate rotation speed *X*_1_, the servo rotation speed *X*_2_ and the interaction term (*X*_1_ *X*_2_) between suction plate rotation speed and servo rotation speed had a highly significant effect on the orientation qualification rate. The ACOA *X*_3_,and the interaction term (*X*_2_ *X*_3_) between the servo rotation speed and the ACOA had a significant effect on the orientation qualification rate. The interaction term(*X*_1_ *X*_3_) between suction plate rotation speed and the ACOA had non-significant effect on orientation qualification rate. The primary and secondary order of factors affecting the orientation qualification rate are suction plate rotation speed, servo rotation speed, and ACOA. After removing the non-significant factors, the regression equations of the factors with qualification rate were established as in Equation 13.


(13) $$Y_{1}=89.17-2.95X_{1}+1.89X_{2}+1.76X_{3}-2.83X_{1}X_{2}-2.53X_{2}X_{3}-2.89X_{1}^{2}-5.01X_{2}^{2}$$

Suction plate rotation speed *X*_1_ and servo rotation speed *X*_2_ had a highly significant effect on root drop rate. the interaction term (*X*_1_ *X*_2_) between suction plate rotation speed and servo rotation speed, and the interaction term (*X*_1_ *X*_3_) of suction plate rotation speed and ACOA had a significant effect on the root drop rate. the interaction term (*X*_2_ *X*_3_) of servo rotation speed and ACOA had a non-significant effect on the root drop rate. The primary and secondary order of factors affecting the orientation qualification rate are servo rotation speed, suction plate rotation speed, and ACOA. After removing the non-significant factors, the regression equations of the factors with drop rate were established as in Equation 14.


(14) $$Y_{2}=9.68+1.69X_{1}+1.74X_{2}-1.25X_{3}-1.45X_{1}X_{2}-1.39X_{1}X_{3}+1.50X_{2}^{2}$$

#### 3.2.2 Response surface analysis

**Figure 15:**
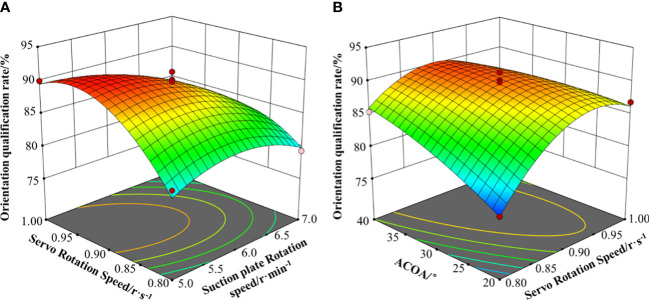
Test bench for orientation transplanting device. 1. root suction device; 2. orientation actuator; 3. orientation control system; 4. PC; 5. target detection model running interface.

As shown in **Supplementary Table 2**, the interaction term (*X*_1_ *X*_2_) between suction plate rotation speed and servo rotation speed and the interaction term (*X*_2_ *X*_3_) of servo rotation speed and ACOA have a non-negligible effect on the orientation qualification rate. **Figure 15** shows the response surface plot of the two interaction terms affecting the orientation qualification rate. As shown in **Figure 15A**, the qualification rate first increases and then decreases with the increase of servo rotation speed and suction plate rotation speed. When the servo rotation speed is less than 0.95 r/s and the suction plate rotation speed is less than 6 r/min, the qualification rate increases with the increase of servo rotation speed and suction plate rotation speed. The increase of servo rotation speed and suction plate rotation speed increases the efficiency of the orientation actuator, and the cumulative error of the programme decreases, which is conducive to the improvement of the qualification rate. The servo rotation speed and suction plate rotation speed continue to increase, the interference of the orientation actuator on the root increases, and the centrifugal force on the root increases, which reduces the qualification rate. As shown in **Figure 15B**, as the ACOA and the servo rotation speed increase, the qualification rate first increases and then decreases. When the servo rotation speed is lower, the qualification rate increases with the increase of the ACOA; when the servo rotation speed is higher, the increase of the ACOA decreases the growth of the qualification rate.

**Figure 16:**
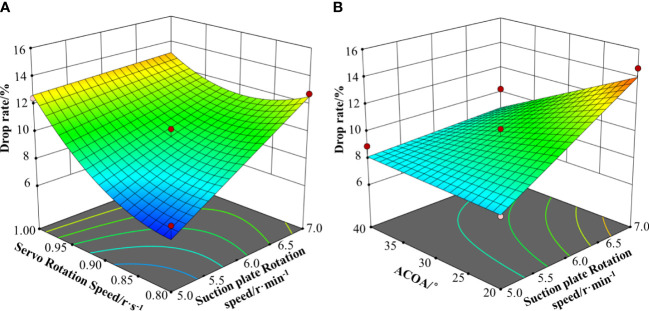
Influence of interactive factors on the root drop rate. **(A)** Y1=f(X1, X2, 0); **(B)** Y2=f(X1, 0, X3).

**Figure 16** shows the response surface plot of the two interaction terms affecting the root drop rate. As shown in **Figure 16A**, the drop rate increases as the servo rotation speed and suction plate rotation speed increase. When the servo rotation speed is lower, as the drop rate increases with the increase of suction plate rotation speed; when the servo rotation speed is higher, the increase of suction plate rotation speed decreases the increase of drop rate. When the suction plate rotation speed is greater than 6.5 r/min, the drop rate first decreases and then increases as the servo rotation speed increases. This indicates that there exists an optimal servo rotation speed that minimises the drop rate when the device is operating in high speed. As shown in **Figure 16B**, as ACOA and the suction plate rotation speed increase, the qualification rate first increases and then decreases. When the suction plate rotation speed is lower, ACOA has less effect on the drop rate; when the suction plate rotation speed is higher, the drop rate increases with the increase of ACOA. When ACOA is lower, the suction plate rotation speed has less effect on the drop rate; when ACOA is higher, the drop rate increases with the increase of suction plate rotation speed.

#### 3.2.3 Parameter optimisation

Reasonable matching between parameters is the key to improve the operation performance. To accurately obtain the optimal parameter combination of each factor, the quadratic regression model established was optimally solved using Design-Expert software. In order to achieve a significant reduction of the drop rate, the weight of the root drop rate in the model is set to be 70%, and the weight of the orientation qualification rate in the model is set to be 30%, and the solution is carried out by using the set objective function, as shown in Equation 15.


(15) $$\left\{ \begin{array}{l} \max Y_{1}(X_{1},X_{2},X_{3})\\ \min Y_{2}(X_{1},X_{2},X_{3})\\ \;s.\;t.\left\{ \begin{array}{c} 5\text{r}/\text{min}\leq X_{1}\leq 7\text{r}/\text{min}\\ 0.8\text{r}/\text{s}\leq X_{2}\leq 1\text{r}/\text{s}\\ 20{}^{\circ}\leq X_{3}\leq 40{}^{\circ} \end{array}\right. \end{array}\right.$$

The optimisation results show that the operation performance reaches the best predicted value when the suction plate rotation speed is 5.73 r/min, the servo rotation speed is 0.86 r/s, and ACOA is 35°. At this time, the orientation qualification rate was 90.86% and the root drop rate was 6.86%.Three validation tests were conducted under these parametric conditions to verify the accuracy of the optimisation results. The test results were averaged. The validation results showed that the orientation qualification rate was 89.87% and the root drop rate was 6.57%, which were basically consistent with the optimisation prediction results. All the test results met the requirements of *Panax notoginseng* root transplanting.

## 4 Discussion

Orientation transplanting of *Panax notoginseng* roots plays an important role in the growth and field management of *Panax notoginseng*. Early researchers developed the semi-automatic transplanting device to replace manual transplanting, which was used to improve the transplanting efficiency (Huang et al., 2023). However, at the root splitting stage, it is needed to manually adjust the root position and place it into the root dropping device. (Zhang J. et al., 2020; Zhang K. et al., 2020). With the increase of labour cost, this type of operation is not conducive to the development of *Panax notoginseng* industry. Furthermore, orientation transplanting devices based on robotic arms are currently only adapted to stationary greenhouse crop transplanting operations (Yang et al., 2018; Liu et al., 2021), and are not controlled in field operations. The transplanting quality and service life of the robotic arm are seriously affected by the uneven road surface and machine-induced vibration. Mature transplanting robots are applied in static situations, and the transplanting efficiency is limited. Lai et al. (2021) and Qin et al. (2022) successively developed automated devices for orientation transplanting of *Panax notoginseng* roots, but suffered from the problems of high breakage rate and low efficiency. In this background, this paper designed a new orientation transplanting device based on the negative pressure adsorption principle and machine vision technology for the low-loss and high-efficiency transplanting of *Panax notoginseng* roots. The experimental results of this paper were compared with the research progress, as shown in **Supplementary Table 3**, the orientation qualification rate increased by 4.2%, and the operating efficiency increased by 23.9%. The root surface damage after transplanting was significantly reduced. The proposed device provides an efficient and accurate solution for achieving consistent root orientation. This research has practical implications for improving transplanting efficiency in the cultivation of various root crops, leading to enhanced agricultural productivity and yield.

It is worthy of note that the current device has some defects. In the experiment, we found that the initial suction posture of the root would affect the transplanting performance. When the root’s centre deviated from the suction hole centre, the drop rate would increase. In future work, the control of the suction posture of the root should be the research focus. In addition, the current device is in the laboratory stage. In field experiments, the detection performance of the model may be disturbed by the environment. In the subsequent work we will use new detection models or improve the current model to enhance the detection performance of the model.

## 5 Conclusions

1. This paper proposed a orientation transplanting device for *Panax notoginseng* roots based on YOLOv5. The automatic orientation and orderly delivery of roots are realised based on negative pressure adsorption and machine vision technology. The orientation method based on machine vision can adapt to the root transplanting operation with shape differences. The orientation method and device proposed in this paper provide ideas for the orientation transplanting of root crops such as *Panax notoginseng*.
2. A detection model for *Panax notoginseng* root cut-main root position was constructed based on YOLOv5s. The detection results show that the precision rate of the model is 94.2%, the recall rate is 92.0%, and the average detection precision is 94.9%; meanwhile, the video detection FPS of the model is 27.13Hz, and the image detection FPS is 42.12Hz.
3. The single-factor experiment and Box-Behnken experiment were carried out on an self-designed test platform. The experimental results were optimised using an objective optimisation algorithm. The optimisation resulted in a suction plate rotation speed of 5.73 r/min, a servo rotation speed of 0.86 r/s and an ACOA of 35°. The theoretical orientation qualification rate under this parameter combination is 90.86% and root drop rate is 6.86%. And the optimisation results were verified. The validation results show that the average value of orientation qualification rate of orientation transplanting device is 89.87%, and the average value of root drop rate is 6.57%. The experimental results are generally consistent with the optimization results, and meet the requirements of orientation transplanting of *Panax notoginseng* roots.

## Data availability statement

The raw data supporting the conclusions of this article will be made available by the authors, without undue reservation.

## Author contributions

QL: Funding acquisition, Resources, Validation, Writing – original draft. YW: Writing – original draft, Writing – review & editing. YT: Writing – original draft, Writing – review & editing. WS: Writing – original draft, Writing – review & editing.
