# Supplementary material for: Design and experiment of Panax notoginseng root orientation transplanting device based on YOLOv5s
Source: Front Plant Sci. 2024 Mar 8;15:1325420. doi: 10.3389/fpls.2024.1325420 (PMC10957537; doi:10.3389/fpls.2024.1325420)
Supplement: Supplementary file 1 [file DataSheet_1.docx]

**Supplementary Tables**

**SUPPLEMENTARY TABLE 1.** Experiment design and results

| Number | Factors | | |  | Index | | |
| --- | --- | --- | --- | --- | --- | --- | --- |
|  | *X*_1_ | *X*_2_ | *X*_3_ |  | *Y*_1_/% | *Y*_2_/% | |
| 1 | -1 | -1 | 0 | 81.13 | | | 7.24 |
| 2 | 1 | -1 | 0 | 79.34 | | | 12.77 |
| 3 | -1 | 1 | 0 | 89.98 | | | 12.46 |
| 4 | 1 | 1 | 0 | 76.87 | | | 12.18 |
| 5 | -1 | 0 | -1 | 85.33 | | | 7.76 |
| 6 | 1 | 0 | -1 | 81.96 | | | 14.65 |
| 7 | -1 | 0 | 1 | 90.45 | | | 8.93 |
| 8 | 1 | 0 | 1 | 85.14 | | | 10.27 |
| 9 | 0 | -1 | -1 | 77.45 | | | 11.65 |
| 10 | 0 | 1 | -1 | 86.87 | | | 14.14 |
| 11 | 0 | -1 | 1 | 85.37 | | | 6.09 |
| 12 | 0 | 1 | 1 | 84.69 | | | 12.89 |
| 13 | 0 | 0 | 0 | 90.17 | | | 8.67 |
| 14 | 0 | 0 | 0 | 89.46 | | | 10.23 |
| 15 | 0 | 0 | 0 | 87.23 | | | 9.26 |
| 16 | 0 | 0 | 0 | 89.87 | | | 8.44 |
| 17 | 0 | 0 | 0 | 91.35 | | | 8.93 |

**SUPPLEMENTARY TABLE 2. Variance analysis of the regression model.**

| Source | Orientation qualification rate | | |  | | Drop rate | | |
| --- | --- | --- | --- | --- | --- | --- | --- | --- |
|  | Sum of squares | Degree of freedom | *F* value | *P* value | Sum of squares | Degree of freedom | *F* value | *P* value |
| Model | 334.45 | 9 | 16.84 | 0.0006** | 93.47 | 9 | 9.83 | 0.0032** |
| *X*_1_ | 69.50 | 1 | 31.50 | 0.0008** | 22.71 | 1 | 21.50 | 0.0024** |
| *X*_2_ | 28.58 | 1 | 12.95 | 0.0088** | 24.22 | 1 | 22.92 | 0.0020** |
| *X*_3_ | 24.64 | 1 | 11.17 | 0.0124* | 12.55 | 1 | 11.88 | 0.0107* |
| *X*_1_ *X*_2_ | 32.04 | 1 | 14.52 | 0.0066** | 8.44 | 1 | 7.99 | 0.0255* |
| *X*_1_ *X*_3_ | 0.9409 | 1 | 0.4264 | 0.5346 | 7.70 | 1 | 7.29 | 0.0307* |
| *X*_2_ *X*_3_ | 25.50 | 1 | 11.56 | 0.0114* | 4.64 | 1 | 4.40 | 0.0743 |
| *X*_1_^2^ | 33.73 | 1 | 15.29 | 0.0058** | 1.69 | 1 | 1.60 | 0.2467 |
| *X*_2_^2^ | 103.40 | 1 | 46.86 | 0.0002** | 8.53 | 1 | 8.07 | 0.0250* |
| *X*_3_^2^ | 4.78 | 1 | 2.17 | 0.1846 | 1.85 | 1 | 1.75 | 0.2271 |
| Residual | 15.45 | 7 | - | - | 7.40 | 7 | - | - |
| Lack of fit | 6.35 | 3 | 0.9311 | 0.5035 | 5.44 | 3 | 3.72 | 0.1184 |
| Pure error | 9.10 | 4 | - | - | 1.95 | 4 | - | - |
| Total sum | 349.90 | 16 | - | - | 100.87 | 16 | - | - |

Note: * indicates a significant effect (*P* <0.05) and ** indicates a highly significant effect (*P* <0.01).

**SUPPLEMENTARY TABLE 3.** Performance comparison with existing devices

| Index | device in this paper | Device (Lai et al., 2021) |
| --- | --- | --- |
| orientation qualification rate(%) | 89.87 | 85.67 |
| Numbers of root transplanted per minute | 57 | 46 |

**Supplementary Figures**


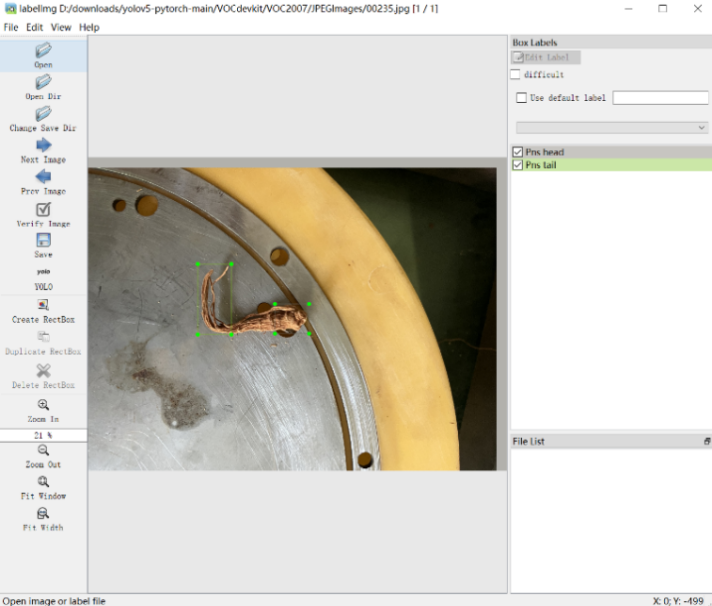


**SUPPLEMENTARY FIGURE 1.** Labeling interface.

**SUPPLEMENTARY FIGURE 2.** Training loss curve.
